# Outcomes for Primary Central Nervous System Lymphoma from a Single Institution

**DOI:** 10.3390/hematolrep17060055

**Published:** 2025-10-24

**Authors:** Sruthi Dontu, Jacob Boccucci, Michael Chahin, Amany Keruakous, Anand Jillella, Jorge Cortes, Vamsi Kota, Locke Bryan, Ayushi Chauhan

**Affiliations:** 1 Medical College of Georgia (MCG), Augusta University, Augusta, GA 30912, USA; 2 Department of Medicine, Wellstar MCG Health Medical Center, Augusta University, Augusta, GA 30912, USA; jboccucci@augusta.edu; 3 Summit Cancer Care, Savannah, GA 31405, USA; chahinmk@gmail.com; 4 Department of Hematology and Oncology, Georgia Cancer Center, Wellstar MCG Health Medical Center, Augusta University, Augusta, GA 30912, USA; akeruakous@augusta.edu (A.K.); ajillella@augusta.edu (A.J.); jorge.cortes@augusta.edu (J.C.); vkota@augusta.edu (V.K.); lbryan@augusta.edu (L.B.); 5 MD Anderson Cancer Center, Texas Medical Center, The University of Texas, Houston, TX 77030, USA; achauhan2@mdanderson.org

**Keywords:** primary CNS lymphoma, outcomes, elderly patients, high-dose methotrexate-based combination chemotherapy

## Abstract

**Background**: Primary central nervous lymphoma (PCNSL) is a rare, aggressive, non-Hodgkin’s lymphoma. Outcomes are poor with standard induction of high-dose methotrexate (HD-MTX)-based regimens and consolidation. We present retrospective data from the Georgia Cancer Center. **Methods**: A single retrospective chart review was conducted on all PCNSL patients from 2013 to 2023 to assess for various factors influencing care. **Results**: Of a total of 38 PCNSL patients, 6 died and 2 were lost to follow-up prior to therapy initiation, leading to a total of 30 patients for analysis. The median age was 62.3 (21–82 years). One patient had HIV/AIDS. Two patients were on immunosuppression for either kidney transplant or multiple sclerosis (MS). The HIV and MS cases were Epstein-Barr Virus (EBV)-positive. Completion of ≥six cycles of induction was predictive of response. **Conclusions**: PCNSL remains an area of high unmet need. Recent studies have shown that HD-MTX-based therapy and autologous stem cell transplantation afterwards leads to improved outcomes regardless of age; however, non-relapse mortality is important to consider. Our data from a primarily elderly and sub-rural cohort reiterate the efficacy of combination chemoimmunotherapy and impact of induction cycle number on response, regardless of age. A multidisciplinary approach and targeted agent maintenance should be considered to improve outcomes in the elderly.

## 1. Introduction

### 1.1. Background

Primary central nervous system lymphoma (PCNSL) is a rare and aggressive malignancy, specifically a non-Hodgkin’s lymphoma, which occurs exclusively in the central nervous system (CNS). Specifically, PCNSL involves pathology within the CNS parenchyma, the dura and leptomeninges, the cranial nerves, the spinal cord, and/or the intraocular compartment. The vast majority of PCNSL cases are of diffuse large B-cell lymphoma (DLBCL) histology [1].

PCNSL has an annual incidence of approximately 0.5 per 100,000 cases per year in the United States. Immunodeficiency is an important risk factor for PCNSL which is considered an AIDS-defining illness. Given the recent rise in highly active antiretroviral therapy (HAART) use, the incidence is increasing more in elderly patients both overall and in the immunocompetent subgroup [1]. A national series of PCNSL in Europe and the United States estimate a prevalence of patients older than 60 years to be between 60 and 70% [2]. Thus, barring epidemiologic trends due to immunodeficiency disorders, PCNSL is now primarily a disease of the immunocompetent older patient (age > 60 years). Large population studies in the United States have also shown an increase in the PCNSL rate of 1.7% per year in people older than 65 years during the period 1992–2011 [3,4]. One nationwide multi-center population-based study of 1002 immunocompetent newly diagnosed PCNSL patients found that 43% of these patients were over the age of 70 years [5]. A more recent single-center retrospective study analyzing PCNSL patients between 2022 and 2024 reported a median age of 65 years [6]. This increased incidence may be attributed to a number of factors, including but not limited to treatment advances in immunodeficiency disorders, increasing life expectancy of the general population and global aging, and diagnostic/treatment advances in PCNSL.

### 1.2. Rationale and Knowledge Gap

Outcomes for PCNSL in general are poor, with a five-year overall survival (OS) of 30% with standard induction of high-dose methotrexate (HD-MTX)-based regimens followed by a consolidative strategy, and an OS of 1.5 months if left untreated [1]. While OS in PCNSL has gradually improved in younger patients, OS in the elderly has not changed in almost five decades. An American nationwide report showed that PCNSL survival for patients >70 years old has remained at approximately six to seven months since the 1970s even though the median OS of all patients doubled from 12.5 to 25 months in the same period [7]. This indicates that the survival benefit remains limited to patients under the age of 70. Studies on elderly sub-rural populations are also limited.

The lack of improvement in survival in elderly patients has been postulated to be due to challenges in standardizing optimal therapeutic strategies in this age group. Diagnostic delays exacerbated by pre-existing cognitive impairments in the elderly may delay onset in patients presenting with more advanced or debilitating disease. HD-MTX-based combination chemotherapy regimens are considered standard induction treatment for PCNSL patients. Elderly patients often have a high incidence of medical comorbidities, poor baseline performance status (PS), and increased effects of drug toxicity that may prohibit usage of these standard regimens. More specifically, impaired renal function can impair metabolism and clearance of MTX, thus increasing treatment toxicity effects, which are further exacerbated by low PS [8]. A recently published retrospective study demonstrated that a lower age correlated with better response and survival rates in those receiving more intensive chemoimmunotherapy. Compared to patients aged between 65 and 75 years, patients over the age of 75 showed a two-year PFS of 7% lower and an OS of 20% lower [9]. Other studies have reiterated high rates of treatment-related toxicity in the elderly with HD-MTX regimens. In one study, 7% of PCNSL patients required ICU-level care and 40% required reductions in first-cycle therapy dosages [10]. Interestingly, a large prospective nationwide cohort of >1000 newly diagnosed PCNSL patients showed that elderly patients were exposed to risk of early death in the first six months after diagnosis. More than 50% of these early deaths were associated with comorbid complications and treatment-related toxicity, exacerbated by precarious health and poor neurological conditions [5]. One recent single-center PCNSL study found that patients had a decent prognosis if they were <65 years, had a performance status of under 2 at diagnosis, and received either intensive or moderate chemotherapy [11]. Another recent cohort study of immunocompetent PCNSL (corpus callosum) patients found that a majority of patients had baseline abnormal neuropsychological findings, including in global cognitive efficiency, memory, and executive function, which was postulated to only recover partially despite treatment response [12]. Given these findings, there is still no current standardized approach for the treatment of PCNSL in elderly patients.

### 1.3. Objective

Given the lack of specific evidence-based consensus in improving outcomes in elderly patients with PCNSL, we present retrospective data for an elderly, sub-rural cohort from our institutional experience over the last decade, with recommendations for improving outcomes.

## 2. Materials and Methods

### 2.1. Patients

We conducted a single-center retrospective analysis on all patients diagnosed with PCNSL at the Georgia Cancer Center from 1 January 2013 to 1 January 2023. A total of 38 patients were initially identified. Eligibility criteria included a diagnosis of PCNSL with a brain lesional biopsy and/or radiographic evidence, as well as presence of follow-up MRIs. Exclusion criteria included death prior to start of therapy and loss to follow-up. Out of the identified patients, 6 died and 2 were lost to follow-up, leading to a total cohort of 30 patients. All but one patient had DLBCL histology, with that one patient receiving treatment on a presumptive diagnosis made by imaging alone given the emergent need for treatment.

Multiple variables were extracted from the electronic medical record, including demographic factors, baseline comorbidities, baseline organ function, induction regimen, number of induction cycles completed, MTX levels at various intervals, end-of-induction (EOI) performance status (PS), median follow-up, median duration of remission, and status at last follow-up. Demographic factors included age at diagnosis, sex, and ethnicity. Age was stratified into <65 and ≥65 years. Sex included two categories: male and female. Ethnicity included six categories: African American, Asian, Caucasian, Hispanic, Native American, or other. Comorbidities were considered at time of diagnosis, including hypertension (HTN), hyperlipidemia, type 2 diabetes mellitus (T2DM), previous stroke/cerebrovascular accident (CVA) history, coronary artery disease (CAD), atrial fibrillation, and if patients were currently on systemic anticoagulation. Baseline HIV status of patients at presentation was also included. Organ dysfunction was evaluated by laboratory values. Lactate dehydrogenase (LDH) levels were included as part of prognostic scores. Hepatitis B and C, as well as baseline creatinine clearance (CrCl), were determined prior to initiation of therapy. Neurological symptoms were also analyzed, including if patients had cerebrospinal fluid (CSF)-positive disease and if seizures were present on initial presentation.

### 2.2. Induction and Consolidation

We analyzed both induction and consolidation treatment regimens. Four different induction regimens were analyzed: HD-MTX; combination high-dose methotrexate, temozolomide, and intravenous rituximab (MTR); combination rituximab, methotrexate, procarbazine, and vincristine (R-MVP); single-agent rituximab (R). Outcomes included response to induction therapy, which was determined by follow-up MRI findings. Responses were classified as complete response (CR), complete response with incomplete count recovery (CRi), partial response (PR), stable disease (SD), or progressive disease (PD). Additional metrics analyzed with regards to induction therapy included completion of ≥ six or < six cycles of induction, MTX levels following induction therapy, median MTX level at 24 h following induction therapy, and the mean discharge MTX level. The end-of-induction (EOI) ECOG performance score was analyzed to determined correlation to response rates. Consolidation therapy included non-myeloablative chemotherapy, myeloablative chemotherapy, and whole-brain radiation therapy (WBRT). Maintenance therapies considered in the patient population included MTX, temozolomide, lenalidomide, Bruton tyrosine kinase inhibitors, and other chemotherapy. Treatment toxicity was analyzed during both induction and consolidation therapy, specifically looking at renal and liver dysfunction and myelosuppression.

### 2.3. Statistical Analysis

Categorical variables were presented as frequencies and percentages, and group comparisons were performed using Fisher’s exact test. Continuous variables were summarized using medians and ranges and compared across induction responses using Kruskal–Wallis one-way analysis of variance (ANOVA). Generated *p*-values were two-sided, with a significance level of 0.05. Statistical analysis was run using IBM SPSS Statistics Version: 29.0.0.0.

### 2.4. Outcome Measures

We analyzed treatment regimens, both induction and consolidation, and the impact on patient care. Four different induction regimens were analyzed: HD-MTX; combination high-dose methotrexate, temozolomide, and intravenous rituximab (MTR); combination rituximab, methotrexate, procarbazine, and vincristine (R-MVP); single-agent rituximab (R). Response rates were determined based on induction therapy and categorized as complete response (CR), complete response with incomplete count recovery (CRi), partial response (PR), stable disease (SD), or progressive disease (PD). Response rates of induction therapy were determined only if patients completed at least six cycles of treatment versus those who did not. Additional metrics analyzed with regards to induction therapy included MTX levels following induction therapy, analyzing the median MTX level at 24 h following induction therapy, and the mean discharge MTX level. The end-of-induction (EOI) ECOG performance score was analyzed to determined correlation to response rates. Progression-free survival (PFS) and overall survival (OS) were not calculated given that the study was not powered for detecting statistical significance in these measures and had a relatively small sample size of 30 patients.

Consolidation therapy included non-myeloablative chemotherapy, myeloablative chemotherapy, and whole-brain radiation therapy (WBRT). Maintenance therapies considered in the patient population included MTX, temozolomide, lenalidomide, Bruton tyrosine kinase inhibitors, and other chemotherapy. Treatment toxicity was analyzed during both induction and consolidation therapy, specifically looking at renal and liver dysfunction and myelosuppression.

## 3. Results

The study cohort consisted of 30 PCNSL patients (Table 1). The median age was 62.3 (21–82 years), and 55% of the cohort was male. Patients identified as non-Hispanic white (77%), African American (10%), Asian (7%), Hispanic or Latino (3%), and multiracial (3%). One patient, the youngest of the cohort at age 21 years, had HIV/AIDS. Two patients were on immunosuppression, one for diseased donor kidney transplant and the other for multiple sclerosis (MS). Two of three patients diagnosed with PCNSL in their 30s were among these immunosuppressed individuals. Both the HIV/AIDS and MS patients tested positive for Epstein Barr Virus (EBV) on quantitative PCR testing. None of the patients had hepatitis B or C. Many patients had comorbidities with 81% of patients having at least one of HTN, T2DM, history of CVA or CAD, and/or atrial fibrillation. A total of 19% of patients had impaired renal function (CrCl < 60 mL/min).

**Figure 1:**
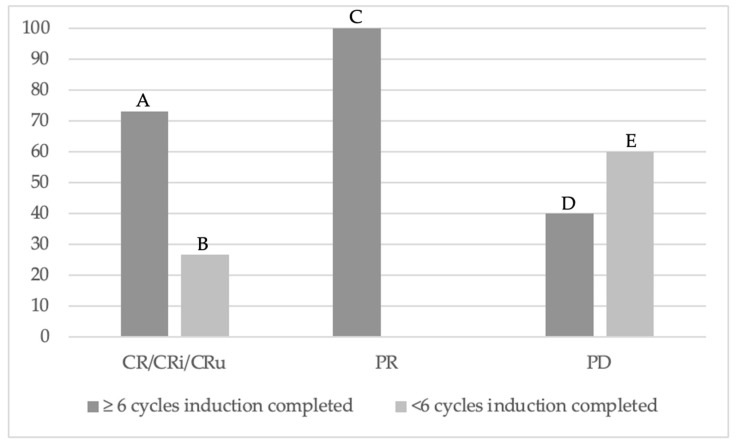
PCNSL outcomes based on number of induction cycles completed. Out of those who achieved CR, 73% completed ≥six cycles of induction (A) and 27% completed <six cycles (B). Out of those who achieved PR, 100% completed ≥six cycles of induction (C). Out of those who experienced PD, 40% completed ≥six cycles (D), while 60% completed <six (E) (*p* = 0.029). CR: complete response; CRi: complete response with incomplete count recovery; CRu: complete response unconfirmed; PD: progressive disease; PR: partial response.

Several patterns emerged when stratified by best treatment response at EOI (Table 2). The median follow-up period was 12 months, with a range of 0 to 66 months. Baseline characteristics, including age, sex, HIV status, positive CSF, creatinine clearance, and elevated LDH, were not statistically significant predictors of response. A total of 15 patients (45%) achieved CR, 5 (16%) achieved PR, and 10 (39%) exhibited PD. Completion of ≥six cycles of induction was associated with a statistically significant better response. Out of those who achieved CR, 73% completed ≥six cycles of induction and 27% completed <six cycles. Out of those who achieved PR, 100% completed ≥six cycles of induction. Out of those who experienced PD, 40% completed ≥six cycles, while 60% completed <six (*p* = 0.029). A majority of those with PD completed < six cycles (Figure 1). A lower MTX level at discharge was also associated with a better response; however, the peak MTX level did not correlate with response, and the sample size was too small to discriminate between discharge MTX thresholds of <0.05 vs. 0.1 vs. >0.15. Four patients (13%) had seizures and all exhibited PD. All patients without seizures responded, and seizures at presentation were associated with a statistically significant worse response to induction. All responders maintained an EOI ECOG of 0–2, and a poorer EOI ECOG was associated with a worse response to induction.

Out of all patients, 17% received monotherapy with HD-MTX and 3% received monotherapy with rituximab. All responders received HD-MTX-based combination chemo-immunotherapy. The median number of cycles of induction completed was seven for those demonstrating CR/CRi/Cru, seven for those demonstrating PR, and three for those demonstrating PD. The median MTX dose intensity was 6800 mg/m^2^ for those demonstrating CR/CRi/Cru, 6750 mg/m^2^ for those demonstrating PR, and 7179 mg/m^2^ for those demonstrating PD. MTX dose intensities by specific induction regimen were also noted. Nine patients required dose reductions overall, with five (33%) in the CR/CRi/CRu category, two (40%) in the PR category, and two (10%) in the PD category (Table 2). Reasons for dose reductions included either induction toxicity and/or delayed MTX clearance. Only eight (27%) patients received consolidative therapy. Seven received non-myeloablative therapy, out of whom two received additional WBRT. Additionally, one patient received WBRT alone for consolidation.

Descriptively, hematologic complications were more frequent than non-hematologic complications (66% versus 34%), with regards to induction treatment toxicities. Grade 1 toxicities were more frequent overall, with 11% more of them found in patients aged ≥65 years versus in patients aged <65 years. Additionally, 10% more of Grade 4 toxicities were found in patients aged ≥65 years versus in patients aged <65 years (Table 3).

## 4. Discussion

Our study highlights key insights into the management of an elderly, sub-rural cohort with PCNSL, emphasizing the efficacy of current chemo-immunotherapy regimens based on the performance status of elderly patients. This is essential, as PCNSL treatment remains challenging, especially in elderly patients who often experience greater toxicity and treatment-related complications.

### 4.1. Institution-Specific Factors Impacting Care in Our PCNSL Cohort

Our academic center has a relatively wide catchment area of a sub-rural population given academic-community outreach efforts within the institution [14]. Most patients in our cohort were seen by a single lymphoma provider, and since 2021, by two lymphoma providers who also performed patient transplants. This was critical for consistent evaluation of transplant candidacy. Since 2022, all PCNSL patients were seen jointly with a neuro-oncologist on the same day or through concurrent appointments, thus emphasizing a collaborative approach. Our current data set, however, cannot show improvement in outcomes due to the relatively small sample size. Given consistency in lymphoma provider evaluation, most patients received MTR, barring recent usage of MATRIX (MTR, cytarabine, thiotepa, rituximab).

### 4.2. PCNSL Outcomes Stratified by Age

Recent evidence from the IELSG43 and MARTA studies supports the use of HD-MTX-based combination chemo-immunotherapy with autologous stem cell transplantation (ASCT) afterwards, demonstrating improved outcomes across different age groups [15,16]. However, non-relapse mortality remains a significant concern, especially in older patients who may not tolerate such intensive regimens. In our cohort, the HD-MTX-based approach achieved meaningful responses, reaffirming its efficacy. Notably, the number of induction cycles appeared to play a critical role in the response rates, suggesting that tailored induction strategies may optimize outcomes in older patients. Our experience also highlights that elderly patients should not be excluded from induction regimens based on age alone but rather individually assessed for whether treatment risk is worth the benefit. In our study, all patients were referred for transplant evaluation but were deemed unsuitable candidates given performance status concerns and efficacy of the chemoimmunotherapy regimens. Furthermore, considering the impact of non-relapse mortality, most patients who received consolidation received non-myeloablative therapy, which is less intensive in nature with only two receiving additional WBRT. In different series and clinical trials, where the objective response rate (ORR) to treatment (chemotherapy and/or radiotherapy) according to age group was available, the elderly group exhibited a lower CR rate, which was reported to be approximately 30–60% versus 50–70% for younger patients [2]. In our study, there was no statistically significant difference in outcomes based on age, likely due to the small sample size. With regard to immunodeficiency, one patient in our cohort had HIV/AIDS and two patients were on immunosuppression. All three patients were on the younger end of the age range, with the former in their 20s and the latter two in their 30s. Given that immunodeficiency is a significant risk factor for PCNSL, it is considered an AIDS-defining illness, and both the HIV and MS cases were positive for EBV. In these cases, PCNSL likely developed as sequelae from underlying immunosuppression [17]. All three patients had variable treatment regimens and responses. The patient with MS received MTR induction, the kidney transplant patient received rituximab monotherapy for induction, and the HIV patient received HD-MTX alone for induction. None of these patients received consolidative therapy. With regard to outcomes, the MS patient showed complete response, while the latter two showed progressive disease. It is notable that of the three patients, the MS patient received seven cycles of induction, while the latter two received two cycles of induction. It is reasonable to postulate that induction cycle number may correlate with response to PCNSL induction treatment even in the younger, immunosuppressed population. Further studies, particularly multi-center ones with larger and more heterogenous immunosuppressed groups, are warranted to explore this impact further and develop approaches towards tailored treatment strategies in these populations.

### 4.3. Impact of Racial and Ethnic Characteristics on PCNSL Outcomes

There are a few epidemiological studies detailing the impact of racial and ethnic characteristics on PCNSL outcomes. One large SEER study comparing survival rates amongst various racial and ethnic groups found that black patients had twice the incidence of PCNSL than white patients in the age group of 20–49 years while the opposite was true in those aged 50 years and older. The influence of HIV presentation on different racial and ethnic groups, in addition to age groups, leading to PCNSL was discussed in [18]. More recent retrospective studies assessing racial and ethnic disparities in PCNSL found statistically significant differences in survival between Hispanic and non-Hispanic patients, both cohorts of which were majority white. This was postulated to be due to diagnosis at younger age and residence closer to hospitals providing treatment [19,20]. Our study comprised a majority of non-Hispanic white patients. Sample sizes of the various racial and ethnic groups were not large enough to allow for meaningful conclusions to be drawn regarding outcomes and care. Further studies with larger sample sizes that are able to better explore the influence of racial and ethnic characteristics on disease presentation, access to care, and therapeutic outcomes in PCNSL are warranted. Specifically, median distance from treatment facilities, comorbidities, and median survival time could be determined to assess for possible contributing factors. Nevertheless, it is important to consider racial and ethnic disparities in care when treating patients with PCNSL.

### 4.4. Treatment Toxicity and Performance Status as Predictors of PCNSL Outcomes in the Elderly

Given that our cohort chiefly comprised elderly patients, several factors related to age emerged as important predictors of outcomes. Elderly patients, as a whole, have a lower functional status at the time of diagnosis, which is an independent prognostic factor in PCNSL. They have more cognitive impairments at diagnosis than those in other age groups. Data from the French LOC network study show a higher proportion of cognitive impairments at the time of PCNSL diagnosis in patients older than 60 years (65% versus 48%, *p* < 0.001) [5]. Older age is also associated with increased comorbidities, which may increase the risk of therapy-induced toxicity [2]. A primary challenge observed in this study was the high rate of treatment discontinuation, often due to performance status deterioration, death from disease, or treatment-related toxicity. For example, one patient receiving MTX at a dose intensity of 8000 mg/m^2^ was not able to complete the seventh induction cycle due to poor performance status concerns. Moreover, dose reductions were observed in 30% of patients overall, often due to either poor tolerance to regimen, hematologic and non-hematologic toxicities like liver enzyme elevation and cytopenias, and delayed MTX clearance post-induction cycle. Per our descriptive results of induction toxicities, Grade 1–4 hematologic and non-hematologic toxicities were more frequent in those aged equal to or above 65 years than in those aged below 65 years.

As reported above, all our PCNSL patients since 2022 were seen by a neuro-oncologist in addition to a lymphoma provider, allowing for greater collaboration, but impact on outcomes could not be assessed due to our relatively small sample size. These findings underscore the need for a more tailored approach that accommodates the frailty and unique needs of elderly patients. A multidisciplinary care model that includes physical therapy, nutrition support, and close collaboration with neurology and geriatrics may help optimize functional status and potentially improve treatment tolerability and outcomes. Such a team-based approach may also aid in addressing the adverse effects that disproportionately impact older and frail patients. Additionally, specific scales can be used to determine treatment risk versus benefit in elderly patients. In a pilot study, Schorb et al. used the Cumulative Illness Rating Scale–Geriatric (CIRS-G) score as an eligibility criterion for PCNSL treatment in addition to age and ECOG status amongst others [21]. This would be a helpful tool to use for PCNSL treatment determination, particularly if standardized. Similar approaches can be developed as standardized measures for determination of induction treatment cost–benefit analysis in the elderly PCNSL population. Future studies could pilot the integration of geriatric assessment tools in prospective data collection to more objectively guide therapy in elderly PCNSL patients. This could be a methodology for future institutional protocols.

### 4.5. Maintenance Options for Elderly Patients with PCNSL

One limitation of this study was the inability to reliably assess effectiveness of various consolidative regimens, given that only eight patients received consolidation. According to the CALGB 502020 study, chemo-immunotherapy of MT-R followed by etoposide-cytarabine (EA) consolidation was found to have comparable outcomes to whole-brain irradiation [22]. While the MARTA study found that HCT-ASCT is feasible and efficacious in selected elderly patients, a retrospective study by the French LOC reported that only around 2% of patients over the age of 60 received consolidation with HCT-ASCT, suggesting that the transplant approach in the elderly is highly dependent on the institution and local practice [5]. In our study, no patients received HCT-ASCT as consolidation, although evaluation of transplant candidacy was consistent, given treatment by one or two lymphoma providers who also performed transplants as mentioned above. Given that HCT-ASCT applied to the general elderly PCNSL population at large comes with toxicity issues, including myelosuppression, exacerbation of medical comorbidities, and potential for cognitive impairment, alternative approaches to consolidation therapy warrant exploration. Targeted agents, such as Bruton tyrosine kinase (BTK) inhibitors, have emerged as promising maintenance strategies in PCNSL. These agents focus on inhibiting BTK, a protein involved in the B-cell receptor/NF-kB signaling pathway that promotes the proliferation of malignant B cells. Initial studies provided evidence for ibrutinib in PCNSL, with one phase I clinical trial using ibrutinib monotherapy in relapsed/refractory (R/R) PCNSL, showing an overall response rate (ORR) of 77%, a median PFS of 4.6 months, and a median OS of fifteen months [23]. While ibrutinib was one of the first FDA-approved BTK inhibitors, many studies indicate that around 19–49% of patients discontinued ibrutinib, and off-target binding has been postulated as a cause. Next-generation BTK inhibitors are expected to bring improved tolerability due to improved selectivity for the BTK protein [24]. Tirabrutinib, one second-generation BTK inhibitor, was the first to be globally approved for R/R/PCNSL, although its effects in combination with other HD-MTX-based regimens are still being explored in an ongoing trial [25]. A few studies have shown promising results of zanubrutinib, a second-generation BTK inhibitor, in combination with other chemotherapy regimens [26,27,28,29]. One such study included a prospective study exploring the effects of a combination regimen of zanubrutinib and high-dose cytarabine in patients with R/R/PCNSL, which found an ORR of 75% and a PFS of 5.6 months [29]. A clinical trial investigating zanubrutinib as monotherapy for R/R PCNSL and SCNSL is currently underway [30]. In our cohort, one patient who received post-induction maintenance with zanubrutinib achieved complete remission, which has now been sustained for approximately 16 months. Other second-generation BTK inhibitors explored for PCNSL include acalabrutinib and orelabrutinib. Other BTK inhibitors have been reported, including TL-895, M7583, and DTRMWXHS-12, for which studies are ongoing and further research required to evaluate their roles in PCNSL [31]. The data and outcomes on BTK inhibitors discussed above support the rationale for exploring these drugs as targeted maintenance therapies and consolidation alternatives in order to reduce toxicity risks in older patients and potentially offering a safer yet effective therapeutic pathway.

### 4.6. Role of Assessing Discharge Methotrexate Levels Post-Induction

Another limitation of this study was the inability to assess the effect of discharge MTX threshold levels on outcomes. Methotrexate level on discharge is an area of discussion in patients being treated with high-dose methotrexate. These patients require close monitoring for adverse events, including nephrotoxicity, mucositis, myelosuppression, and neurotoxicity. In a recent study exploring updated discharge criteria for HD-MTX, decreasing the threshold from 0.05 to 0.1 was found to decrease length-of-stay, which subsequently resulted in decreased cost per admission without increasing complication rates [32]. In our study, the relatively small sample size made it difficult to distinguish between discharge MTX thresholds of below 0.05, 0.1, and above 0.15. Generalizability may also be limited given the small sample size and single-center nature of the study limited to patients in Georgia.

### 4.7. Study Limitations

To summarize, this study has several limitations, which confine its scope, contextualize its findings, and guide insight into areas for future research. One major limitation is the relatively small sample size inherent to a retrospective single-institutional study, which prevents the assessment of outcomes based on factors like consistency in lymphoma evaluation and collaborative visits with both lymphoma providers and neuro-oncologists. It also limits the power of the study in examining impactful outcomes, like PFS and OS. Furthermore, the study was specific to an institutional experience in Georgia, which limits its overall generalizability. This also contributed to the inability to assess the effect of discharge MTX threshold levels on outcomes, as discussed above, where the small sample size made it difficult to distinguish between thresholds of below 0.05, 0.1, and above 0.15. Future studies may warrant multi-center collaboration and/or pooled analyses that can raise the power further through expanded sample sizes. This would further validate findings and significantly improve external validity. Another limitation of the study was the inability to reliably assess the impact of various consolidative regimens on outcomes, as a minority of the patients received consolidation. Future directions may include more tailored studies exploring the impact of specific BTK inhibitors on outcomes as they gain traction as consolidative strategies in PCNSL and further integrate molecular profiling into care. Other limitations of the study include those that are modifiable in future research endeavors, such as toxicities and prospective follow-up and monitoring over a period of time. Treatment-related complications were not assessed in detail in this study, particularly with regards to consolidation. This could be expanded in future studies to encompass various systems and assess for any difference between hematologic and non-hematologic complications of various induction and consolidation regimens, including impact on outcomes. Prospective research design would also enable collection of more real-time standardized data, further strengthening the evidence of causality. These studies could incorporate the utilization of geriatric assessment tools to better assess individualized treatment needs in elderly PCNSL patients. Real-world registry studies could also be performed to obtain more diverse and representative populations, further increasing the generalizability of outcomes. Overall, the above approaches for future directions would be able to address the therapeutic and supportive care gaps identified in this study.

## 5. Conclusions

Overall, our study reports outcomes in a real-world, elderly, sub-rural cohort of PCNSL patients who are underrepresented in trials. These patients received a consistent induction regimen and were treated mostly by a single lymphoma provider who also performed transplants, with more recent incorporation of collaborative neuro-oncology visits. The data recapitulate those seen in larger datasets and highlight the ongoing unmet need in PCNSL. The association between induction cycles completed and treatment response underscores the role of systemic chemotherapy regimens in PCNSL patients regardless of age. It highlights the need for tailored treatment strategies in elderly patients to include systemic chemotherapy based on a global assessment of fitness of elderly patients, with particular emphasis on maintenance strategies, in order to minimize toxicity and maximize efficacy. The use of zanubrutinib in a patient currently in complete remission also highlights the role of next-generation BTK inhibitors in consolidative regimens and warrants future studies exploring this in more detail. This approach may help to bridge the gap in current PCNSL management. Future directions include larger, multi-center analyses, prospective research design, and/or real-world registry studies, thus enabling the stronger generalizability of outcomes to PCNSL populations.

## Figures and Tables

**Table 1:** Baseline characteristics of PCNSL patients. CR: complete response; CRi: complete response with incomplete count recovery; CRu: completion response unconfirmed; CSF: cerebrospinal fluid; HIV: human immunodeficiency virus; LDH: lactate dehydrogenase; PD: progressive disease; PR: partial response. An asterisk next to the *p*-value indicates statistical significance.

N = 30	CR/CRi/CRu	PR	PD	*p*-Value
N (%)	15 (45)	5 (16)	10 (39)	
Age				0.89
<65 years	6 (40%)	2 (40%)	5 (50%)	
≥65 years	9 (60%)	3 (60%)	5 (50%)	
Gender				0.61
Male	7 (47%)	3 (60%)	6 (60%)	
Female	8 (53%)	2 (40%)	4 (40%)	
Baseline LDH				0.88
>ULN	5 (33%)	2 (40%)	5 (50%)	
<ULN	9 (60%)	2 (40%)	5 (50%)	
Unavailable	1 (7%)	1 (20%)	0	
Baseline HIV status				0.49
Positive	0	0	1 (10%)	
Negative	15 (100%)	5 (100%)	9 (90%)	
Baseline creatinine clearance				0.23
≥60	14 (93%)	3 (60%)	7 (70%)	
<60	1 (7%)	2 (40%)	3 (30%)	
Baseline CSF positive				0.56
Yes	2 (13%)	1 (20%)	2 (20%)	
No	8 (53%)	1 (20%)	5 (50%)	
Unavailable	5 (33%)	3 (60%)	3 (30%)	
Seizures at presentation				<0.001 *
Yes	0	0	4 (40%)	
No	15 (100%)	5 (100%)	6 (60%)	

**Table 2:** Induction-related characteristics. ECOG: Eastern Cooperative Oncology Group; HD-MTX: high-dose methotrexate; MTR: methotrexate, temozolomide, and rituximab; PS: performance status; R: rituximab; R-MVP: rituximab, methotrexate, vincristine, procarbazine. One patient received zanubrutinib and remained in CRu. An asterisk next to the *p*-value indicates statistical significance.

N = 30	CR/CRi/CRu	PR	PD	*p*-Value
Induction Regimen				0.83
HD-MTX Alone	1 (7%)	0	4 (40%)	
MTR	11 (73%)	5 (100%)	4 (40%)	
R-MVP	3 (20%)	0	1 (10%)	
R	0	0	1 (10%)	
Number of treatment cycles completed				0.029 *
≥6	11 (73%)	5 (100%)	4 (40%)	
<6	4 (27%)	0	6 (60%)	
24-h peak MTX level Median (Range)	37.7 (1.88–490.9)	33.99 (23–149.46)	42.51 (0.99–212.34)	0.31
Mean Discharge MTX level				0.013 *
<0.05	4 (27%)	0	4 (40%)	
0.05–0.1	5 (33%)	4 (80%)	5 (50%)	
>0.1	4 (27%)	0	1 (10%)	
Unavailable	2 (13%)	1 (20%)	0	
End of induction PS (ECOG)				0.002 *
0–2	12 (80%)	4 (80%)	2 (20%)	
>2	3 (20%)	1 (20%)	8 (80%)	
Median follow-up (months) (range)	Total cohort: 12 months (0–66)			
	19	30.5	4.5	
Median duration of remission (months) (range)	Total cohort: 16 months (0–103)			
	25	40	1	
Status at last follow-up				
Remission	13 (87%)	3 (60%)	8 (80%)	
Relapse	2 (13%)	2 (40%)	2 (20%)	

**Table 3:** Induction toxicities of cohort summarized and presented as n (%). Grades 1–4 for each individual toxicity were defined based on the National Cancer Institute’s Common Terminology Criteria of Adverse Events, version 5.0 [13].

Induction Toxicities	Total	Grade 1	Grade 2	Grade 3	Grade 4
Number of individual below toxicities	82 (100%)	29 (35%)	25 (31%)	18 (22%)	10 (12%)
Non-hematologic	28 (34%)	13 (16%)	6 (7%)	9 (11%)	0 (0%)
Hepatotoxicity	20 (24%)	8 (10%)	4 (5%)	8 (10%)	0 (0%)
Nephrotoxicity	8 (10%)	5 (6%)	2 (2%)	1 (1%)	0 (0%)
Hematologic	54 (66%)	16 (20%)	19 (23%)	9 (11%)	10 (12%)
Neutropenia	15 (18%)	2 (2%)	5 (6%)	2 (2%)	6 (7%)
Thrombocytopenia	16 (20%)	9 (11%)	4 (5%)	1 (1%)	2 (2%)
Anemia	23 (28%)	5 (6%)	10 (12%)	6 (7%)	2 (2%)
Age ≥ 65 years	51 (62%)	19 (23%)	13 (16%)	10 (12%)	9 (11%)
Age < 65 years	31 (38%)	10 (12%)	12 (15%)	8 (10%)	1 (1%)

## Data Availability

The original contributions presented in this study are included in the article material. Further inquiries can be directed to the corresponding author.
